# Oral Cavity Squamous Cell Carcinoma: An Update of the Pharmacological Treatment

**DOI:** 10.3390/biomedicines11041112

**Published:** 2023-04-07

**Authors:** Martina Imbesi Bellantoni, Giacomo Picciolo, Igor Pirrotta, Natasha Irrera, Mario Vaccaro, Federico Vaccaro, Francesco Squadrito, Giovanni Pallio

**Affiliations:** 1 Department of Biomedical, Dental, Morphological and Functional Imaging Sciences, University of Messina, Via C. Valeria, 98125 Messina, Italy; martinaimbesi@gmail.com; 2 Department of Clinical and Experimental Medicine, University of Messina, Via C. Valeria, 98125 Messina, Italy; giacomo.picciolo@unime.it (G.P.); igor.pirrotta@unime.it (I.P.); nirrera@unime.it (N.I.); mario.vaccaro@unime.it (M.V.); gpallio@unime.it (G.P.); 3 Department of Dermatology, University of Modena and Reggio Emilia, Via Del Pozzo, 41124 Modena, Italy; federico.vaccaro92@gmail.com

**Keywords:** oral cavity squamous cell carcinoma, OCSCC, OCSCC pharmacological treatment

## Abstract

Oral cavity squamous cell carcinoma (OCSCC) represents a serious health and socio-economic problem in different geographical areas of the world. It is characterized by a high rate of mortality, recurrence and metastasis. Despite the therapeutic strategies implemented for its management and resolution, currently the survival estimate for locally advanced disease is about 50%. The available therapeutic options comprise surgery and pharmacological treatment. Recently, an increased emphasis has been placed on the drugs that might be of benefit in this life-threatening disease. Therefore, the aim of this present review was to offer a general survey of the current available pharmacological treatment for OCSCC. The PubMed database was used to retrieve the papers using “OCSCC” as the search terms. We limited our search to the last 5 years to give a more updated and recent picture of the state of the art, including preclinical and clinical investigations. We found that 77 out of 201 papers were on the surgical treatment of OCSCC, 43 out of 201 focused on the radiotherapy and 81 out of 201 underwent evaluation for the aim of our review. We excluded the case reports, editorial letters, observational studies and papers written in languages other than English. A total of 12 articles were included in the final review. Our results showed that nanotechnologies use to enhance the efficacy of anticancer drugs such as: cisplatin, paclitaxel, cetuximab, EGFR antagonists, MEK1/2 and immune check inhibitors combination could have promising anti-cancer activity. However, the paucity of available data on drugs suggests the urgent need to improve the pharmacological armamentarium for OCSCC treatment.

## 1. Introduction

Oral Cavity Squamous Cell Carcinoma (OCSCC) is the most common type of cancer in Head and Neck Cancer (HNC), ranking 16th place throughout the world. Therefore, it is catalogued among the most aggressive malignant tumors due to its metastatic action and to its high recidivism rate [1]. OCSCC has an incidence that varies between 1% and 4% in the western world, with the higher prevalence in unindustrialized Asian countries where it is associated with a high mortality rate [2]. The global burden of OCSCC is 3.90/100,000, whereas the mortality rate of oral cavity tumors is around 1.94/100,000 people [3]. Given the strong global impact, the urgency of addressing OCSCC as a global health priority is evident [4]. Recent epidemiology data published by the National Cancer Institute (NCI), showed that the overall survival rate for OCSCC in a 5-year period is 63% with a range between 83% in early stages and 38% in advanced stages [5,6,7]. The histological typing of the OCSCC is heterogeneous. To date, there are six histological variants recognized by the OMS: (i) The verrucous carcinoma, a variant that usually occurs as an outgrowth of prolonged tobacco chewing. It seems to be a less-aggressive form over the more common conventional SCC. (ii) The squamous basaloid cell carcinoma, a particularly rare form of tumor that has moderately pleomorphic basaloid cell population and fewer squamous cells. The epithelium overlooking the tumor appears to be generally dysplastic, and it majorly targets the tongue. Moreover, it often appears at later stages and is defined by a high incidence of local relapse and regional lymph node metastases, and, for these reasons, it is considered to be a quite aggressive cancer. (iii) The spindle cell carcinoma, a variant that targets mainly the aerodigestive tract, characterized by an aggressive course and a fast growth. (iv) The adeno-squamous carcinoma, histologically defined by both some of the adenocarcinomas and the SCC features. (v) The adenoid squamous carcinoma, a variant which usually occurs on sun-exposed skin but is somewhat found inside the oral cavity [8,9]. These tumors are usually classified according to five different aspects: deep invasive margins, ratio of keratinized cells, nuclear polymorphism, invasiveness grade and the inflammatory infiltrate’s intensity as previously described by Bryne at al. [10].

Risk factors depend on the population’s lifestyles. Major risk factors for OCSCC are: tobacco, alcohol, betel quid chewing and viruses [11].

The smoke produced by cigarettes contains various harmful elements, such as nitrosamines, benzopyrenes and aromatic amines that increase the risks of carcinogenesis, thus weakening the oral region’s immunity. Other types of tobacco use, such as chewing tobacco, is associated with an increase in oral cavity cancer risk. In 2007, the AIRC clearly stated that there are an adequate number of proofs confirming that tobacco smoke and nasal tobacco are cancerous in light of their production of oxygen and nitrogen free radicals that affect antioxidant defense mechanisms in the oral epithelium [12].

Alcohol consumption is highly correlated with the development of oral cavity tumors and with the presence of various impurities like nitrosamine and mycotoxin, which are particularly carcinogenic. Alcohol abuse also significantly augments the effects of other carcinogens like tobacco. Thus, the possibility of developing upper aero-digestive tract cancer is exponentially enhanced in people who both smoke and drink, indicating a synergistic effect. In this regard, the cytochrome p450 (CYP2E1), which is one of the principal xeno-metabolizing enzymes involved in the activation of N-nitrosamines associated with tobacco, is inducible by ethanol. The initial phase of ethanol metabolism is mediated by the alcohol dehydrogenase that produces acetaldehyde, a cytotoxic molecule that fosters both the production of free radicals and the hydroxylation of the DNA base. The alcohol dehydrogenase type 3 genotypes are associated with rapid oxidation of alcohol in acetaldehyde, massively predisposing patients to oral cancer [13].

Betel-quid is composed of sun-dried and aged areca nut mixed with slaked lime and occasionally with various spices, incorporated inside a betel pepper leaf. Chewing Betel (also referred to as areca nut) is estimated to be the fundamental etiological factor in the development of oral submucosal fibrosis. Nonetheless, the use of betel quid that includes both tobacco and areca nut has been found to be 15 times more cancerous than quid usage without tobacco which still has a potential for malignancy that is between 1 and 4. If chewed, betel quid releases reactive oxygen species that cause highly detrimental effects at the mucosa of the oral cavity level. Production and release of reactive oxygen species (ROS) take place in alkaline conditions throughout autoxidation of the areca nut’s polyphenols inside the betel quid chewer’s saliva. ROS, then, can be involved in the tumor-starting process in two ways: by inducing genotoxicity and genetic mutation or rather by attacking salivary proteins and then causing a structural leveled mutation of the oral mucosa that causes weakening and facilitates the penetration of other environmental toxicant agents [14,15,16].

The presence of a correlation between viruses and oral cancer has been demonstrated. The viruses most involved in oral cancer transformation are the human papillomavirus (HPV), herpes group virus, adenovirus and hepatitis C virus [17]. Among these, HPV and herpes are the most studied and, to date, are both considered the most frequently associated viruses involved with human oral cancer. HPVs are epitheliotropic DNA viruses, which can induce hyperplastic, papillomatous and verrucous squamous cells in stratified squamous epithelia of skin and mucous membrane injuries. Nearly 100 different types of HPV genotypes exist, but a particular regard must be paid to HPV-16 and HPV-18, both strongly associated with malignancy and often described as malignant oncogenic genotypes or rather high-risk genotypes [18,19,20,21].

The tongue, the oral cavity’s floor and inferior lip are the sites with the highest probability of developing OCSCC [22]. This cancer is usually diagnosed in its later stages, and this is the reason why it diminishes survival chances, despite therapeutic strategies. The first evaluation of the tumor is based on inspection and on a careful palpation of the nodal drainage area. This approach offers to clinicians a clinical staging (cTNM) that is a highly accurate instrument, straightforward and universally accepted for the staging process to stratify patients, to select the best therapy and to evaluate the therapeutic alternatives [23].

Nowadays, elective therapy is surgical resection followed or preceded by either chemotherapy or radiotherapy. Nevertheless, in recent years new therapeutic approaches which require either monotherapy or the use of a combined pharmacological treatment have been investigated to enhance patients’ living standards [24]. To date, surgical resection is still considered to be elective therapy for OCSCC treatment. According to the data provided by the literature, the tongue is the most affected site, followed by the gingiva, the buccal mucosa and the mouth floor, while the lip remains the least affected anatomical area. It is of paramount importance to perform a scrupulous staging based on a clinical and physical examination using radiographic insights. Modern imaging techniques such as TC and RMN are, today, the mainly recommended techniques for a loco-regional disease evaluation. OCSCC requires a widening of resection margins that pose an aesthetic and functional problem for the patient. For oral carcinoma, the three-dimensional resection margins up to 1 cm are considered reasonable. After the primary tumor’s enucleation, the so-called “restitutio ad integrum” is considered necessary for the affected anatomical area, and, therefore, the next step is to proceed with reconstructive surgery, starting with a skin graft up to a microvascular free flap. The “free flap” transfers are the most widely used oral reconstruction technique. Moreover, it is well known that the state of the neck lymph nodes has the most important prognostic value in OCSCC. A percentage of just above 50% of patients affected by oral carcinoma in its early stages will turn out to have a clinically node-negative neck. Nevertheless, about 20% to 30% of patients will show microscopically detectable lymph node metastases by post-dissection histological neck examination. It is important to underline how the likelihood of neck metastasis is significantly higher for the tongue and mouth floor than for the hard palate and gingiva, these latter showing a comparatively low metastasis rate to the neck. Thus, to date, the widely used and accepted treatment for the cervical lymph node metastasis is indeed neck dissection [25,26,27,28,29,30,31,32,33,34,35,36,37,38,39].

Due to the constant progress made with diagnosis and treatment technologies, the survival rate of OCSCC has substantially increased although there is a great challenge for the researcher to reduce the adverse events related to the absence of selectivity and toxicity in drugs that are commonly used nowadays. In fact, after surgery or radiotherapy and chemotherapy, patients could be affected by various local defects and collateral effects such as: disfunctions, malformations and drug resistance, specifically to drugs that may not be tolerable and could cause, in most of the cases, relapse and metastasis, leading to a poor quality of life of the patients. Since the beginning of the 21st century, many drug therapies have been developed against specific cancer genetic targets, including growth factor receptors, key molecules involved in signal transduction or transcription activation, and genes related to tumor cell proliferation, division, invasion, and metastasis. More recently, targeted therapies have been developed in order to identify suitable drugs against specific carcinogenic sites that could massively improve patients’ quality of life, raising their survival rate up to 5 years [13]. Therefore, in light of all these observations, the purpose of this review was to provide an update on pharmacological treatments available for OCSCC.

## 2. Research Strategy

The PubMed database was used to retrieve the papers using as a search term “OCSCC” in order to identify the most appropriate studies and exclude other types of head and neck cancer localized at different anatomical sites. Moreover, we limited our search to the last 5 years to give a more updated and recent picture of the state of the art pharmacological treatment of OCSCC.

The search generated 201 papers: 77 out of 201 papers were on the surgical treatment of OCSCC, 43 out of 201 focused on the radiotherapy and 81 out of 201 underwent evaluation carried out by two authors well trained in the treatment of the disease. Regarding the clinical studies, we excluded the case reports, editorial letters and observational studies, concentrating only on clinical trials. The non-clinical studies were divided into in vitro and in vivo investigations. Finally, papers written in languages other than English were excluded.

## 3. Results

A total of 12 articles were included in the final review. Definitive papers were subdivided into in vitro, in vivo, and clinical studies, and we analyzed the retrieved results in accordance with this stratification

### 3.1. In Vitro Studies

#### 3.1.1. Nanographene Oxide Loaded with Doxorubicin

Nanotechnologies represent a great potential to ameliorate and enhance the targeting of cancer cells while avoiding the deleterious appearance of side effects. Tumor tissues, including OCSCC, are acidic with a pH value in the range of 6.5–6.8, which is lower than that of normal tissue (pH ~7.4). Ph-sensitive nanocarriers could significantly improve the bioavailability of anticancer drugs delivered to the tumor site, sparing normal cells from cytotoxicity. Recently a DOX@NGO-BBN-AF750 nanocomposite has been synthesized by the non-covalent bonding method to couple carboxylated NGO with BBN-AF750 and doxorubicin through π–π and hydrogen bonding in order to improve the doxorubicin’s tumor targeting. This nanocomposite achieved fluorescence imaging and controlled drug release in cancer cells, opening up new possibilities for an imaging-guided therapy of OCSCC. In this context, Li et al. explored the cell internalization and antitumor activity in an in vitro model of oral squamous cell carcinoma. They demonstrated that DOX@NGO-BBN-AF750 provides a slow release of doxorubicin from the nanoprobe, thereby avoiding drug degradation and enhancing the half-life. This nanocomposite provides a precise approach for a targeted therapy of OCSCC that, if confirmed by clinical studies, could represent a significant improvement in the pharmacological treatment of OCSCC [40].

#### 3.1.2. Metformin Combined with 4SC-202

Metformin is a low-cost agent, and it has been found to be of great interest for its anticancer action. He et al. showed metformin’s ability to inhibit invasion and migration of squamous cell tumors of the oral cavity. More specifically, this study evaluated the effects of metformin, 4SC-202, a novel selective class I histone deacetylase inhibitor (HDACi) that has previously been reported to inhibit the survival and proliferation of several types of cancer cells including OCSCC, or their combination. Metformin combined with 4SC-202 blunted OSCC cell migration and invasion by suppressing STAT3/TWIST1 signaling [41]. This study is of paramount importance. In fact, metformin is available on the market as a treatment for type II diabetes, and its combination with 4SC-202 could be easily translated into the clinical scenario. Furthermore, the reported synergy between the two compounds is expected to amplify the anticancer activity and minimize the occurrence of side effects.

#### 3.1.3. Procyanidin B2 (PB2)

Procyanidin B2 (PB2) is an important single component of procyanidin that is a common natural crosslinking agent with strong antioxidant capacity. This antioxidant compound promotes autophagy and apoptosis of colorectal cancer cells through phosphatidylinositol-4,5-bisphosphate 3-kinase (PI3K)/protein kinase B (AKT) signaling pathways and exerts cytotoxicity in breast cancer cells. It has been demonstrated that PB2 could inhibit angiogenesis and cell growth in OCSCC through the VEGF/VEGFR2 pathway. More specifically, Sun et al. used different concentrations of PB2 to treat an OSCC cell line (SCC-25), and then they evaluated the viability, apoptosis, invasion and migration of SCC-25 cells and determined the changes of the VEGF/VEGFR2 pathway affected by PB2. They found that PB2 inhibited the VEGF/VEGFR2 pathway and, therefore, suppressed the cell growth and angiogenesis, suggesting the possible use of this compound as an adjuvant therapy in OCSCC patients [42]. This interesting result deserves to be confirmed in preclinical in vivo studies as well as in patients with OCSCC.

#### 3.1.4. Cetuximab and Cisplatinum-Conjugated Gold Nanoparticles

PEGylated nanodrug complexes could be potentially effective in generating high cytotoxicity at low doses, enhancing the radio therapeutic (RT) activity in RT-resistant oral cavity cancer cells. Moreover, they can be conjugated with Cetuximab (CTX) to obtain an active targeted nanodrug complex with active/passive radio sensitizing properties. The main advantages of PEGylated GNP nanodrug complexes include their excellent biocompatibility and their low biological toxicity compared to other traditional agents such as cisplatin together with X-ray irradiation properties. This confers to these nanodrug systems increased efficacy at low doses, thereby potentially reducing the extent of radiation therapy for patients. Sùrer and co-workers synthesized the PNL-conjugated cisplatin and cetuximab complexes as nanodrugs and assessed the efficacy of a combination treatment with radiotherapy in radiotherapy-resistant oral cavity cancer cells. This study demonstrated that PEGylated nanodrug complexes are potentially effective in generating high cytotoxicity at low doses by significantly enhancing radiotherapy activity in RT-resistant oral cavity cancer cells to obtain a targeted active nanodrug complex. These results will provide a valid approach to apply the highly effective nano-radiosensitizer to treat oral cavity cancers and to avoid the occurrence of radiotherapy resistance [43].

#### 3.1.5. Cisplatin + Paclitaxel

Cisplatin and paclitaxel are two of the most widespread drugs used to treat oral cancer. Cisplatin acts through the formation of intra-strand crosslinks with the purine bases in DNA. It is broadly used to treat many cancers, including OCSCC even if acquired resistance to cisplatin often abrogates its therapeutic efficacy. Recent studies suggested that combining cisplatin with other anticancer drugs might overcome this resistance and may provide a new treatment strategy for many cancers.

Paclitaxel is an antimicrotubule agent that binds to microtubules during cell division and induces apoptosis. In several cisplatin-resistant cancer cell lines, cisplatin and paclitaxel combination chemotherapy has been used as a method for modulating cisplatin sensitivity. Choi and colleagues investigated the effects of a combination between cisplatin and paclitaxel on three parental (YD-8, YD-9, and YD-38) and three cisplatin-resistant (YD-8/CIS, YD-9/CIS, and YD-38/CIS) OSCC cell lines using cell proliferation assays and the combination index analysis. They demonstrated that the overexpression of FOXM1 protein induced by cisplatin causes resistance to paclitaxel, which can potentially attenuate the effectiveness of the combination with the two chemotherapeutic agents for oral cancer. In light of these findings, they speculate that patients could benefit from paclitaxel monotherapy considerably more than from a cisplatin–paclitaxel combination therapy as a second-line regimen when the first-line cisplatin regimen has failed [44].

#### 3.1.6. Anlotinib

Anlotinib is a novel oral multitarget receptor tyrosine kinase inhibitor that has been approved as a third-line treatment for refractory advanced non-small-cell lung cancer in light of its capacity to regulate tumor cell proliferation, apoptosis, angiogenesis, migration and invasion. Anlotinib showed a tolerable safety profile in a variety of malignant tumors, such as medullary thyroid cancer, renal cell carcinoma, gastric cancer and esophageal squamous cell carcinoma [45,46]. Recently, Lu et al. investigated the efficacy of anlotinib on HSC-3 human oral squamous cell carcinoma. They demonstrated that anlotinib significantly prevented the spread of HSC-3 cells in a dose-dependent manner, inhibiting their proliferation, viability and migration. Furthermore, they showed that this composite anticancer activity may derive from the inhibition of the PI3K/Akt phosphorylation pathway. This work provides evidence for using anlotinib to treat patients with OSCC even if further in vivo and clinical studies are needed [47] (Table 1).

### 3.2. In Vivo Studies

#### 3.2.1. Nimotuzumab

Nimotuzumab blocks cell proliferation and angiogenesis, boosts natural killer cells, primes dendritic cell maturation and induces cytotoxic T cells. This drug also restores MHC-I expression in tumor cells, hindering one of the EGFR immune-escape ways. Moreover, nimotuzumab is well-tolerated, and, for this reason, its use in combination with conventional therapies could be an important strategy for controlling OCSCC. Aminolevulinic acid (ALA)-based photodynamic therapy (PDT) has been shown as an optional strategy for patients affected by OCSCC. Photochemical reactions during PDT are produced by photosensitizers (PS), tissue oxygen and specific wavelengths of light which can induce reactive oxygen species (ROS)-mediated apoptosis of OCSC cells. He et al., in a paper published last year, evaluated the potential synergy between nimotuzumab and PDT in killing OCSC cells, exploring also their biosafety using a mouse OCSCC xenograft model. They demonstrated that tumor growth in the combined group was significantly slower than in the other groups up to 7 days after treatment. Moreover, three of the five mice that received combined ALA-PDT with nimotuzumab showed a complete remission. These results suggested that a combined treatment of EGFR inhibitors (i.e., nimotuzumab) with ALA-PDT may increase the cure rate in OCSCC patients without increasing toxicity even if further clinical studies will be necessary to confirm these interesting pre-clinical results [48].

#### 3.2.2. Metformin Combined with 4SC-202

In OSCC, high expression of histone deacetylase (HDAC) was associated with poor prognosis, advanced stage, larger tumor size and lymph node metastasis in patients, indicating that HDAC plays a pivotal role in OSCC progress. Therefore, the use of histone deacetylase inhibitors such as 4SC-202 could inhibit the growth and induce the apoptosis of OSCC cells. Several studies have revealed that metformin treatment was associated with lower cancer incidence and that its use can increase oral cancer cell sensitivity to chemotherapeutic drugs, improving the treatment efficacy and lowering toxicity. He et al. demonstrated that combined metformin and 4SC-202 treatment synergistically suppressed the growth and promoted intrinsic apoptosis by accelerating ΔNp63 ubiquitination and degradation of OSCC in vivo. These findings highlighted that a combined treatment of metformin and 4SC-202 could be a promising potential therapeutic strategy for OSCC [49] (Table 2).

### 3.3. Clinical Studies

#### 3.3.1. Trametinib

Trametinib (GSK1120212) is an allosteric MEK1/2 inhibitor having a more extended half-life than past generations of MEK inhibitors. Trametinib is Food and Drug Administration (FDA) approved for use in combination with dabrafenib for incurable BRAF mutant melanoma or also as a single agent. Previous studies demonstrated that trametinib was generally well tolerated. However, trametinib use has not yet been deeply investigated in head and neck squamous cell carcinoma (HNSCC) or specifically in OCSCC. In a recent paper, Uppaluri and colleagues evaluated patients with stage II–IV OCSCC receiving trametinib (2 mg/day, minimum 7 days) prior to surgery. They demonstrated that trametinib administration in patients with OCSCC led to a decrease of Ras/MEK/ERK pathway activation and a reduced CD44 expression. This resulted in a marked clinical benefit indicated by a response, in agreement with the World Health Organization criteria, that was evidenced in 11 out of 17 (65%) evaluable patients (median 46% decrease, range 14 to 74%). Moreover, a partial metabolic response (≥25% reduction in SUVmax) occurred in 6 of 13 (46%) evaluable patients (median 25% decrease, range 6 to 52%). Finally, clinical-to-pathologic tumor downstaging was present in 9 of 17 (53%) evaluable patients. Overall, trametinib resulted in significant reduction in Ras/MEK/ERK pathway activation and in clinical and metabolic tumor responses, resulting in a promising therapeutic option for OCSCC patients [50].

#### 3.3.2. Nivolumab

Nivolumab is a human immunoglobulin G4 PD-1 immune checkpoint inhibitor antibody that blocks PD-1, promoting antitumor immunity that could be effective for the treatment of non-small-cell lung cancer (NSCLC), melanoma, renal cell carcinoma (RCC) and other cancers [51,52,53]. Knochelmann et al., in a single arm phase-II trial, enrolled 12 patients with stage II-IVA OCSCC receiving 3 to 4 biweekly doses of 3 mg/kg nivolumab. They demonstrated that patients treated with presurgical nivolumab therapy had an overall response rate of 33% with a median follow up of 2.23 years and a survival rate around 80%, thus suggesting that nivolumab is safe and well-tolerated. The authors concluded that neoadjuvant nivolumab is safe and well-tolerated and could be proposed as neoadjuvant therapy for OCSCC patients [54].

#### 3.3.3. Nanoengineered Cisplatin

Cisplatin, cis-diamminedichloridoplatinum (II), is an anticancer drug that hinders cancer cell proliferation by the formation of intra-strand crosslinks with the purine bases in DNA. Nevertheless, considered a non-selective targeting drug, both the healthy tissue and the malignant tissue could be affected; thus, various adverse effects such as marked nausea, emesis, ototoxicity, acute nephrotoxicity, myelosuppression and chronic neurotoxicity can hamper its clinical use. In recent years, a new self-adhesive cisplatin transmucosal system PRV111 has been developed and studied to convey cisplatin-loaded chitosan particles (CLPs) to anatomically accessible oral cancers, notably lip, tongue, gum, floor of mouth, gingiva, buccal mucosa, and many other oral cavity areas. PRV111 is a thin, 2-layer, matrix-type, polymeric transmucosal patch, designed to arrange targeted drug delivery and prevent CLP washout from saliva, and it consists of a chitosan matrix layer embedded with CLPs and an impermeable ethyl-cellulose adhesive backing. Each PRV111 topical patch contains 0.5 mg/cm^2^. The system also incorporates a permeation enhancer (PE) that enables the most optimal penetration and absorption of the CLPs unbound from the patch by reversibly opening the tight junctions between the cells. The released CLPs bloat to comparatively 0.5 micron when exposed to moisture, so they can then scatter all atop the porous matrix and then right into the tumor tissue. Goldberg et al. demonstrated that there is no systemic cisplatin exposure after 24 h since these particles are too large to penetrate the vasculature (vasculature pore size is 2–15 nm). Moreover, they evaluated the safety and efficacy of the PRV111 by using various entrenched animal models to study oral mucosal carcinogenesis, demonstrating the superior local retention, efficacy and safety of the CLPs in contrast to either non-encapsulated or intravenous cisplatin administration in all models tested. Furthermore, in a phase 1/2, open-label, single-arm trial, they showed the efficacy (≥30% tumor volume reduction) and safety of neoadjuvant PRV111 with a 69% tumor decrease in ~7 days and an over-87% response rate. Moreover, all secondary endpoints (cisplatin biodistribution, loco-regional control, and technical success) were reached. Finally, no drug-related serious adverse events or locoregional recurrences were reported in 6 months [55]. In conclusion, the integration of PRV111 with current standard of care may have positive effects on the health outcomes and survival of patients with OCSCC.

#### 3.3.4. Camrelizumab + Apatinib

Camrelizumab, a PD-1inhibitor, and apatinib, a VEGFR2 antagonist, have shown promising activity in several types of cancers. New neoadjuvant therapy systems are warranted for the treatment of OCSCC. Ju and colleagues enrolled 20 patients with locally advanced resectable OCSCC receiving three cycles of camrelizumab (200 mg, every 2 weeks) and apatinib (250 mg, once daily) before surgery. The primary endpoints were safety and major pathological response (MPR, defined as ≤10% residual viable tumor cells). Secondary endpoints included a 2-year survival rate and local recurrence rate. They observed that neoadjuvant treatment is well-tolerated, and the MPR rate is 40% (8/20), meeting the primary endpoint. Moreover, all patients with CPS ˃10 achieved MPR. Furthermore, the post-hoc analysis showed an 18-month locoregional recurrence and survival rates of 10.5% and 95%, respectively. Finally, patients treated with neoadjuvant therapy achieving MPR showed more CD4+ T-cell infiltration than those without MPR (*p* = 0.02) and decreased CD31 and ɑ-SMA expression levels [56]. In conclusion, neoadjuvant camrelizumab and apatinib are safe and could be promising for improving outcomes and overall survival in OCSCC patients (Table 3).

## 4. Conclusions

Surgical resection is an elective therapy for the treatment of OCSCC. Radiotherapy represents an additional therapeutic alternative for patients suffering from OCSCC. Pharmacotherapy has also been proven to offer a significant clinical advantage, especially in locally advanced disease. The aim of this review was to analyze the available data in the literature in support of a drug treatment. A total of 11 articles were included in the final review. Nanotechnologies used to enhance the efficacy of anticancer drugs such cisplatin, paclitaxel and cetuximab, EGFR antagonists, MEK1/2 and immune check inhibitors combination showed promising anti-cancer activity. The targets of these new therapies unmask favorable antitumor activity in terms of inhibition of angiogenesis activity, reduction of adverse events, and lowering of the risk of recurrence. However, the lack of robust information on the mutations underlying the pathogenetic and resistance mechanisms hampers the search for innovative pharmacological treatments, and the consequent paucity of available data on new medicines suggests the urgent need to improve the pharmacological armamentarium for OCSCC treatment. Indeed, the data emerging from this review can give promising answers on alternative pharmacological therapeutic choices to conventional chemotherapy; much more promising opportunities will be offered in the near future, which could finally guarantee a better quality of life and a high survival rate in patients with OCSCC.

## Figures and Tables

**Table 1:** Summary of in vitro studies.

Study	Reference	Cell Line	Pharmacological Treatment	Effect
Li et al., 2022	[40]	HS3	DOX@NGO-BBN-AF750	Increase drug half-life. Decrease drug degradation.
He et al., 2020	[41]	HSC6 CAL33	Metformin + 4SC-202	Inhibition of STAT3/TWIST1. Suppression of invasion and migration of OSCC.
Sun et al., 2022	[42]	SCC-25	Procyanidin B2	Inhibition of VEGF/VEGFR2 pathway. Suppress the cell growth and angiogenesis.
Sürer et al., 2021	[43]	UPCI-SCC-131	Cetuximab and Cisplatinum-conjugated Gold Nanoparticles	Increase cytotoxicity. Overcome resistance to radiotherapy.
Choi et al., 2020	[44]	YD-8, YD-9, YD-38, YD-8/CIS, YD-9/CIS, YD-38/CIS	Cisplatino + Paclitaxel	Combination of cisplatin and paclitaxel had an antagonistic effect.
Lu et al., 2021	[47]	HSC-3	Anlotinib	Inhibit PI3K/Akt/Bad phosphorylation and promote apoptosis by activating RAS protein expression.

**Table 2:** Summary of in vivo studies.

Study	Reference	Pharmacological Treatment	Effect
He et al., 2022	[48]	Nimotuzumab	Inhibit cell proliferation and promote apoptosis in OSCC, increasing the cure rate.
He et al., 2019	[49]	Metformin + 4SC-202	Inhibit cancer cell growth and induce intrinsic cell apoptosis through increasing ΔNp63 ubiquitination and degradation.

**Table 3:** Summary of Clinical Studies.

Study	Reference	Pharmacological Treatment	Effect
Uppaluri et al., 2017	[50]	Trametinib	Reduction of Ras/MEK/ERK pathway activation and in clinical and metabolic tumor reponses.
Knochelmann et al., 2021	[54]	Nivolumab	Overall response rate of 33% with a median follow up of 2.23 years.
Goldberg et al., 2022	[55]	Nanoengineered Cisplatin (PRV111)	69% tumor reduction in ~7 days and over-87% response rate. No DLTs or drug-related serious adverse events were reported.
Ju et al., 2022	[56]	Camrelizumab + Apatinib	MPR rate of 40%. At 18 months, 10.5% of locoregional recurrence and 95% of survival rates.

## Data Availability

Not applicable.
